# Increased expression of Toll-like receptors and associated alarmins in temporal arteries of patients with giant cell arteritis

**DOI:** 10.1186/s10020-025-01390-4

**Published:** 2025-11-12

**Authors:** S. Seidlberger, M. Schirmer, G. Wietzorrek, J. A. Jiménez-Heffernan, M. Pardines, M. de las Fuentes Monreal, M. A. González-Gay, S. Castañeda, S. Santos-Sierra

**Affiliations:** 1 Institute of Pharmacology, Medical University of Innsbruck, Innsbruck, Austria; 2 Department of Internal Medicine II, Medical University of Innsbruck, Innsbruck, Austria; 3 Department of Pathology, Hospital Universitario La Princesa, Madrid, Spain; 4 Rheumatology Division, University Hospital La Princesa, Instituto de Investigación Sanitaria-Princesa, IIS-Princesa, Madrid, Spain; 5 Maxillofacial Surgery Division, University Hospital La Princesa, Madrid, Spain; 6 Rheumatology Division, IIS-Fundación Jiménez Díaz, Madrid, Spain; 7 University of Cantabria, Santander, Spain; 8 Cardiovascular Pathophysiology and Genomics Research Unit, School of Physiology, Faculty of Health Sciences, University of the Witwatersrand, Johannesburg, South Africa; 9 Department of Medicine, Universidad Autónoma de Madrid (UAM), Madrid, Spain

**Keywords:** Temporal artery biopsy, Giant cell arteritis, Temporal arteritis, Polymyalgia Rheumatica, Innate immunity, Toll-like receptors

## Abstract

**Background:**

Giant cell arteritis (GCA) is a chronic granulomatous inflammatory disease involving large- and medium-sized arteries. The disease spectrum comprises cranial (C-GCA), extracranial (EC-GCA) and mixed phenotypes. Toll-like receptors (TLRs) in the affected arteries may play an important role in GCA pathogenesis. However, data on TLR and TLR-ligands expression pattern in GCA arteries are lacking.

**Objective:**

To investigate the expression of TLRs and putative ligands in temporal artery biopsies (TAB) from C-GCA, EC-GCA and isolated polymyalgia rheumatica (PMR) patients to establish a link between TLRs, antigen expression, and disease stage. To correlate the plasma levels of identified TLR-ligands with standard inflammatory markers (IL-6, CRP, ESR) in these patients.

**Methods:**

Immunofluorescence staining of TLR2/4/7/8, HMGB-1, SAA, fibrinogen, and p-glycoprotein was performed with TABs of six biopsy proven C-GCA, six EC-GCA, five PMR patients and seven age-matched controls. Association studies among plasma inflammatory markers were done with 139 PMR and 40 GCA patients.

**Results:**

The levels of TLR2/4/7/8 and the alarmins HMGB-1, SAA, and fibrinogen were highly increased in C-GCA TABs in the sites of inflammation and less in EC-GCA TABs. P-glycoprotein was overexpressed in C-GCA TABs. Glucocorticoids or TAK1-inhibitor treatment decreased the fibrinogen- and SAA-mediated IL-6 production in control PBMCs. Plasma levels of SAA and fibrinogen associated strongly with CRP and ESR levels.

**Conclusion:**

TLRs are overexpressed at the site of vascular inflammation in C-GCA and at a lower level in EC-GCA and PMR with negative TAB. Moreover, HMGB-1, SAA, and fibrinogen may serve as disease biomarkers of patients with C-GCA.

**Supplementary Information:**

The online version contains supplementary material available at 10.1186/s10020-025-01390-4.

## Introduction

Giant cell arteritis (GCA) is an inflammatory disease primarily involving medium- and large-arteries that affects people over 50 years. Typically, GCA involves the temporal artery (TA) and other arteries derived from the external carotid artery, and ophthalmic arteries derived from the internal carotid artery which is named as cranial GCA (C-GCA). Some patients present a predominant extracranial symptomatology without cranial ischemic manifestations and are defined as extracranial-GCA phenotype (EC-GCA). Additionally, there is an overlap between cranial ischemic manifestations and extracranial involvement confirmed by imaging techniques (mixed phenotype) (Gonzalez-Gay et al. 2019). The affected arteries show intimal hyperplasia and luminal obstruction, which leads to ischemic manifestations, being blindness one of the most ominous complications (Hayreh 1998; Gonzalez-Gay 2016). Fortunately, the incidence of vision loss has decreased in recent decades (Gonzalez-Gay 2016).

Polymyalgia rheumatica (PMR) is a disease characterized by pain and stiffness in the shoulders, proximal aspects of arms, hip girdle, and neck that may manifest independently or coexist with GCA. There is a significant overlap between these two conditions, with PMR occurring in up to 50% of GCA patients (Gonzalez-Gay 2004). Imaging techniques (e.g. [^18^F]-fluorodeoxyglucose positron emission tomography-computed tomography (PET-CT)) have revealed extracranial large vessel vasculitis (LVV) involvement in up to a third of patients with isolated PMR (Gonzalez-Gay 2023; Gonzalez-Gay 2017), and some studies suggest that the two disorders may represent different poles of the same process (Tomelleri et al. 2023; Dejaco 2017).

Histological examination of temporal artery biopsy (TAB) shows that, at a cellular level, Th-1 and Th-17 cells are recruited to the adventitia-media border by activated resident dendritic cells (Deng 2010). Then, they induce the migration and differentiation of macrophages into multinucleated giant cells (MNGC). These, produce cytokines, growth factors, and proteolytic enzymes that target vascular smooth muscle cells (VSMCs) leading to intimal hyperplasia and, in turn, to stenosis or occlusions with further ischemic events (Weyand 2023).

It has been suggested that the arterial inflammation in GCA may be instigated by a pathogen (e.g. Epstein-Barr virus, varicella zoster virus), although these findings are controversial (Ostrowski 2019). On the other hand, the immune activation may be triggered by danger-associated molecular patterns (DAMPs or alarmins). These host-derived antigens (e.g. SAA, HMGB-1 [high mobility group box-1]) are usually expressed intracellularly, but can be extracellularly released due to cellular damage following stress or inflammatory stimuli. HMGB-1 is localized as a nuclear DNA-binding protein in most cell types (Ugrinova 2009). Yet, extracellular HMGB-1 may activate several receptors (e.g. Receptor of Advanced Glycation End-products (RAGE), TLR2, and TLR4) and stimulate the release of extra-cellular matrix (ECM) proteins (Wang et al. 2015).

Several studies tried to identify antigens specifically overexpressed in the TABs of C-GCA patients, especially in cases where morphological evaluation and hematoxylin/eosin (H&E) staining show inconclusive results. For instance, CD68 staining is more sensitive but less specific for diagnosing GCA than histopathological findings (e.g. presence of MNGC and transmural inflammation (Muniz Castro 2022)). Adding CD3 immunostaining to the standard histology increases sensitivity with a comparable specificity (Ciccia 2018). In contrast, CD83 staining did not improve GCA diagnosis (Slobodin 2007). However, in GCA patients with very recent optic nerve ischemia, PTX3 levels are higher in comparison to controls (Baldini et al. 2012).

The expression of innate immune receptors, such as TLRs, has been shown to be increased in the blood cells of GCA patients (Alvarez Rodriguez et al. 2011). In addition, the expression of TLR2, TLR4, and TLR8 in several arteries of healthy controls was elevated (Pryshchep 2008). However, to the best of our knowledge, TLR protein expression in TABs of GCA patients at the time of diagnosis has not been evaluated so far via immunofluorescence studies. Yet, a recent study using nanostring nCounter gene expression profiling uncovered 31 downregulated and 256 upregulated genes in GCA TABs compared to normal TABS, being some TLRs overexpressed (Ferrigno et al. 2024).

PMR and GCA patients´ blood samples are characterised by increased acute-phase reaction markers (e.g. ESR, CRP, IL-6). High levels of IL-6 correlate with clinical symptoms and glucocorticoid (GC) treatment suppresses IL-6 production. Notably, withdrawal of GC after several months of treatment is followed by an immediate increased IL-6 levels (Roche 1993). Nevertheless, IL-6 has been questioned as a precise marker to monitor patients treated with the IL-6R antagonist tocilizumab (TCZ) and GCs, and additionally, IL-6 blockade influences the levels of other inflammatory markers (Samson et al. 2024).

In this work, we aimed to identify novel antigens that may be overexpressed in the TABs of clinically active C-GCA patients and to determine if they also occur in TAs of patients with EC-GCA and PMR in whom TABs yield negative results. Increased expression of these antigens in patients with GCA and isolated PMR may be useful in diagnosing patients for whom standard H&E staining of biopsies does not yield positive results. Furthermore, we examined whether additional laboratory parameters like fibrinogen and SAA show an association with the routinely analysed inflammatory markers.

## Patients and methods

### Subjects

TABs from six C-GCA patients, six EC-GCA, five PMR, and seven controls were obtained from the Pathology Department, UHLP. Clinical and biological data (Table S1) were recorded at the time of TAB acquisition and updated with the TAB results and final diagnosis. GCA patients fulfilled either the 1990 American College of Rheumatology (ACR) classification criteria (Hunder et al. 1990) or the 2022 American College of Rheumatology/EULAR GCA classification criteria (Ponte et al. 2022a; 2022b). Patients with EC-GCA had a negative TAB and were diagnosed with extracranial LVV by imaging techniques (ultrasound, angio-CT, or PET-CT). Isolated PMR had a negative TAB, no manifestations of cranial or extracranial ischemia, and negative imaging results. None of the PMR patients developed clinical features of GCA during 1-year of follow-up. Controls were defined as age-matched subjects who had undergone a TAB and later received a different diagnosis. PMR patients fulfilled the preliminary classification criteria of PMR proposed by EULAR/ACR (Dasgupta et al. 2012).

For the association analysis, a confirmation cohort was included with data from 139 consecutively recruited PMR and 40 GCA patients (Table S2) who attended a routine examination in the Rheumatology Department, MUI.

### Specimens´ preparation and immunofluorescence of temporal arteries

TABs were obtained from the Pathology archives at UHLP, Madrid, Spain. Sections were cut at 4 µm and mounted on glass slides for staining with H&E. C-GCA patients had biopsy-proven GCA with typical histopathologic features (e.g. transmural inflammation with lymphocytes, macrophages, and MNGC, fragmentation of the internal elastic membrane (IEM), intimal hyperplasia, and luminal narrowing).

The samples were deparaffinised and washed before epitope retrieval, performed by heating twice to 96 °C for seven minutes and cooling down for 30 min. After washing with TBS-T (50 mM Tris-buffered saline pH 7.2, 0.4% Triton X-100) and blocking with TBS-T/5% normal goat serum (NGS) at room temperature for two hours, the primary antibodies (Table S3) were incubated on the slides for 20 h at 4 °C (1:200 in TBS-T/5% NGS). After washing with TBS-T, the secondary antibody was added onto the slides (1:1000 in TBS-T/5% NGS) for two hours at room temperature, followed by washing steps. Hoechst 33,342 (Cell Signaling Technology) was used for the DNA staining (1:100,000 in phosphate-buffered saline) for 60 s. Slides were sealed with mounting medium (Fluoroshield™; Sigma Aldrich; F6182) and images were acquired with a PeconCellVivo microscope and the software CellSens Dimension. Control of the secondary antibodies staining is shown in Figs. S2-3, and by controlling the background fluorescence in the corresponding red, green, and blue channels.

### Quantification of antigens

The relative antigen expression was assessed with ImageJ software (FijiWin64/ImageJ2015). Due to the morphological heterogeneity of the samples, we quantified equivalent areas. Accordingly, we traced two rectangles (same width, 65 µm) extending from the artery lumen until the interior border of the adventitia (comprising tunica intima plus tunica media; Fig. S1). The first rectangle was in every case situated on the broader part of the artery and the second was displaced at an angle of ninety degrees. The mean value (relative intensity) of both surfaces was calculated.

### Cytokine measurement

PBMCs were isolated from whole blood samples using Histopaque®−1077 (Sigma-Aldrich; 10,771). Cells were seeded in 96-well plates (1.2 × 10^5^ cells/well) in RPMI-1640 with 10% Fetal bovine serum (Sigma-Aldrich) and 0.5% Ciprobay (Bayer). After 24 h, the cells were treated with 5Z-7-oxozeanol (Sigma-Aldrich; O9890) or prednisone (Dermapharm GmbH; 201,024) for one hour and stimulated with fibrinogen (Sigma-Aldrich; 341,576-M) or SAA (Sigma-Aldrich; SRP4324). The cytokine released in the supernatant (24 h) was detected by enzyme-linked immunosorbent assay (ELISA) (BioLegend; 430,501) with a plate reader (Victor^2^ ™D, Perkin Elmer) and absorbance at 450 nm. The detection limit for IL-6 was 7.8 pg/ml.

### Statistical analysis

Statistical analysis was performed with GraphPad Prism 10.2.3 (San Diego, CA, USA) after normality testing of the data. In the IF experiments, results were expressed as median value with standard deviation. Comparisons between two groups were done with the Mann–Whitney U test. For the cytokine measurement, the statistical analysis was done by one-way ANOVA followed by Dunnett´s multiple comparison test. *P*-values were reported as significant if *p* < 0.05 (*); *p* < 0.01(**); *p* < 0.001(***); *p* < 0.0001 (****). Associations were determined with Spearman´s rank correlation coefficient. One-tailed *P*-value (*p* < 0.05) was considered statistically significant.

## Results

### Expression of TLRs in C-GCA, EC-GCA, and PMR artery samples

**Fig. 1:**
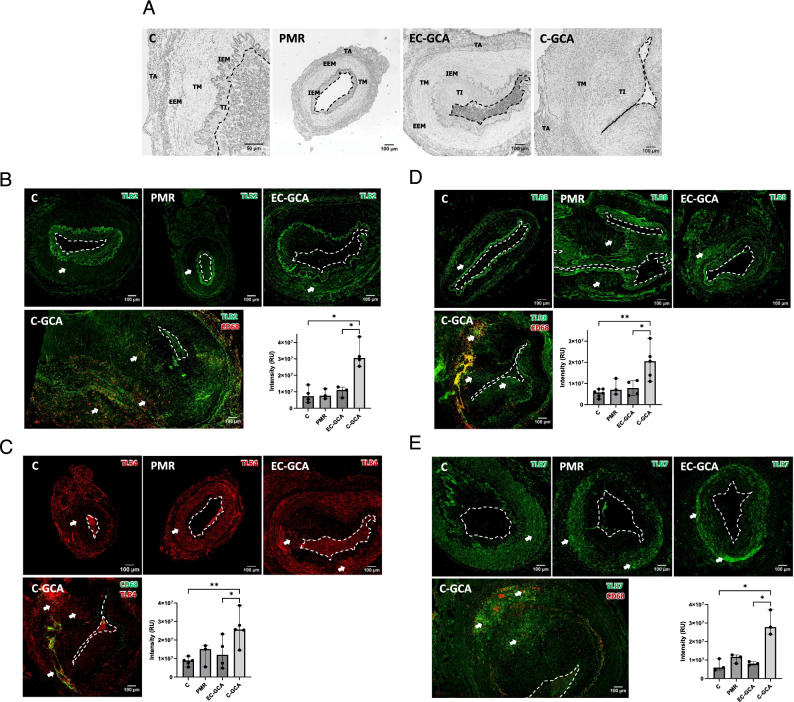
Expression of different TLRs in C-GCA, EC-GCA, PMR patients, and controls. **A** Representative bright field images of C-GCA, EC-GCA, PMR, and control samples (**C**). TA: Tunica adventitia. EEM: External elastic membrane. TM: Tunica media. IEM: Internal elastic membrane. TI: Tunica intima. **B**-**E** TLR2, TLR4, TLR8 and TLR7 immunofluorescence (IF) staining. Arrows point to TAB regions with high expression of the receptors. The graph-bars represent the median and standard deviation of at least three values. The differences significance was assessed with Mann–Whitney U test. *p* < 0.05 (*); *p* < 0.01(**). For improved clarity, the lumen is marked with an open white or black lane. RU: relative intensity units

TLR expression in TABs of GCA patients at diagnosis has not been investigated yet. Thus, we performed immunofluorescence (IF) staining of TLR2, TLR4, TLR7, and TLR8 in TABs from C-GCA, EC-GCA, PMR patients, and controls. Histological evaluation of the TAB specimens (Fig. 1A) demonstrated that C-GCA arteries had IEM destruction and luminal occlusion due to the proliferation and migration of VSMCs into the tunica intima. EC-GCA samples presented a thickened tunica intima in some cases. In contrast, most samples from PMR patients who had no clinical evidence of GCA were morphologically normal.

The expression level of the above indicated receptors was quantified in the media and the intima arterial layers according to the scheme shown in Fig. S1. In the case that high background staining was observed in the IEM (which might lead to misrepresentative results), only the expression in the tunica media was taken into account (i.e. TLR2, TLR8). Additionally, the background staining with the secondary antibodies used was assessed (Figs. S2, 3).

Immunofluorescence staining of TLR2 in TABs is depicted in Fig. 1B. TLR2 is expressed at low levels in the tunica media of controls, PMR and EC-GCA. In contrast, its expression is between three and four times higher in C-GCA TABs where it was found in resident cells and in invading macrophages (CD68^+^ cells). Yet, CD68 cells are mostly located at the media-adventitia border and in the adventitia. Various commercial antibodies that were initially tested in IF experiments rendered negative staining results (Fig. S4).

Next, we investigated the expression of TLR4 (Fig. 1C) which appeared expressed at low levels in the tunica media of controls, and at increasing levels in PMR and EC-GCA samples. It was found markedly overexpressed in C-GCA TABs (3.2 times more than in controls) at the intima-media border, the media, and the media-adventitia border, where invading macrophages are also localized.

TLR8 (Fig. 1D) showed an atypical expression pattern. Contrary to the homogeneous expression of TLR2 and TLR4 in the media layer, TLR8 is expressed in distinct areas in all the samples, C-GCA, EC-GCA, PMR, and controls. In C-GCA patients, a high density of TLR8 was observed at the site of arterial stenosis and in CD68^+^ cells. In addition, TLR8 was localized in smooth muscle cells in the tunica media in control and in the tunica media and tunica intima in GCA samples (Fig. S7). The observed TLR8 expression pattern was confirmed with two different antibodies (Fig. S8). Another endosomal receptor, TLR7, was expressed at low levels in the tunica media of controls, PMR, and EC-GCA samples (Fig. 1E). In contrast, its expression was highly increased (3.8 times) in the intima and media layers and the media-adventitia border of the C-GCA samples, primarily at the stenosis site. CD68 co-staining showed that not all the macrophages co-expressed TLR7.

Increased overexpression of specific TLRs in arterial tissue might indicate which DAMPs may incite or perpetuate the inflammatory reaction.

### Expression of DAMPs linked to GCA inflammation

We focused our investigations on DAMPs previously recognized as ligands for TLR2 and TLR4.

**Fig. 2:**
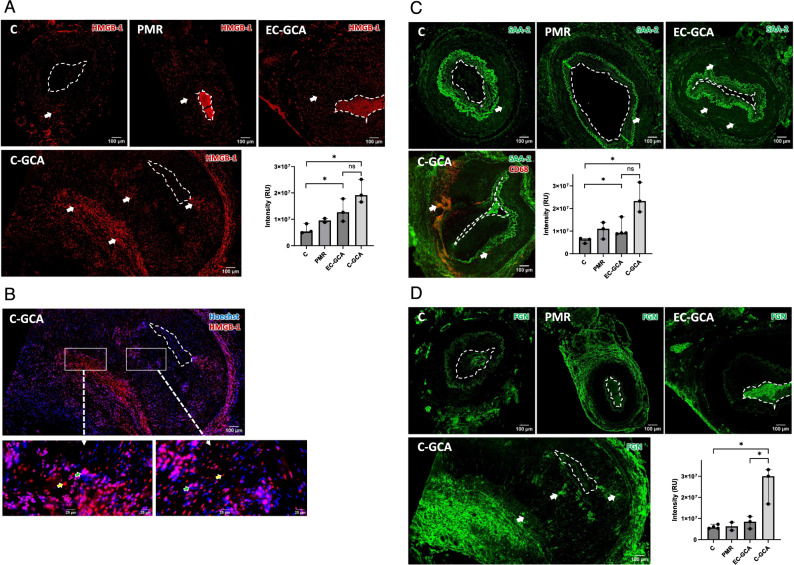
Expression of DAMPs in TABs of C-GCA, EC-GCA, PMR patients and controls. **A** HMGB-1. **B** HMGB-1 plus Hoechst dye for the nuclei staining. HMGB-1 cytoplasmic and ECM expression (red; yellow arrows) nuclear expression (pink; green arrows). **C** SAA. **D** Fibrinogen. Arrows point to TAB regions with high expression of the proteins. The graph-bars represent the median and standard deviation of three values. The differences significance was assessed with Mann–Whitney U test p-values significant if *p* < 0.05 (*); ns: non-significant. For improve clarity, the lumen is marked with an open white lane. RU: relative intensity units

IF staining of HMGB-1, a ligand of TLR2/TLR4, and RAGE (Park et al. 2004; Tian et al. 2007), showed that HMGB-1 was expressed in the media of TABs from controls, and that the level of expression increased gradually from PMR and EC-GCA to C-GCA samples (Fig. 2A). In the latest, extensive patches of HMGB-1 expression can be observed in the media and media-adventitia border.

In physiological conditions, HMGB-1 is located in the nucleus, and it is transported to the cytoplasm or to the ECM following stress conditions or cell death. Thus, TABs were stained with the nuclear dye Hoechst. As shown in Fig. S5, in PMR and EC-GCA samples, HMGB-1 appears mainly in the cell nucleus, as in control samples, but in C-GCA TABs (Fig. 2B) it was detected in the nucleus of some cells, in the cytoplasm, and likely in the ECM.

Circulating SAA is increased in GCA patients in comparison to controls, and it was proposed as a regulator of TA inflammation and angiogenesis (O'Neill et al. 2015). However, its expression in C-GCA TABs has not been investigated. In TABs from PMR and EC-GCA patients, SAA is increased in the tunica media compared to controls (Fig. 2C). In C-GCA TABs, SAA was highly expressed mainly in the intima-media and media-adventitia borders where macrophages were also found.

Fibrinogen degradation products are elevated in the serum of PMR and C-GCA patients before GC treatment (Grau 1984), and extravascular fibrinogen may activate immune cells via TLR4 (Smiley 2001). Following staining with anti-fibrinogen, background expression was observed in the adventitia in PMR, EC-GCA, and control samples, but very low expression was detected in the media layer. On the contrary, in C-GCA samples, expression in the media and media-adventitia border was raised approximately 3 times in comparison to controls (Fig. 2D).

### Expression of p-glycoprotein in C-GCA, EC-GCA, PMR, and control arteries

**Fig. 3:**
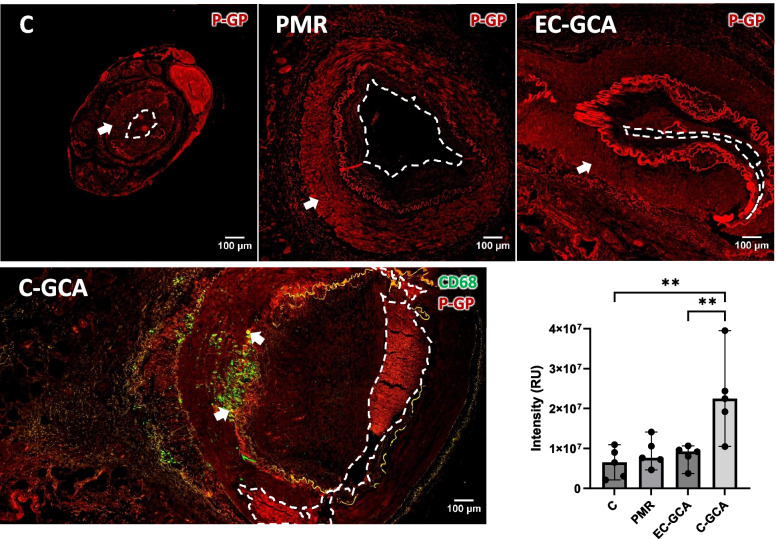
Expression of p-glycoprotein in TABs of C-GCA, EC-GCA, PMR patients and controls. Arrows point to TAB regions with high expression of p-glycoprotein. The graph-bars represent the median and standard deviation of five values. The differences significance was assessed Mann–Whitney U test *p* < 0.01(**). For improved clarity, the lumen is marked with an open white lane. RU: relative intensity units

During follow-up, PMR and GCA patients may experience a reduced response to GCs or suffer from flares after tapering to a lower dose (Castaneda 2022; Alba et al. 2014). One of the possible mechanisms of GC resistance is the cellular overexpression of p-glycoprotein (Smutny 2019), and it has been shown that p-glycoprotein expression is influenced by TLR2 activity in murine and human myeloid cells (Frank et al. 2015). We assessed the levels of p-glycoprotein via IF (Fig. 3), and we observed that it was expressed similarly in the media of controls, PMR, and EC-GCA samples. However, in C-GCA samples, p-glycoprotein expression was significantly increased in the intima and media lamina and at the transitional border, where macrophages were also localized.

### GCs or TAK1 inhibition decrease the fibrinogen- or SAA-mediated IL-6 production

**Fig. 4:**
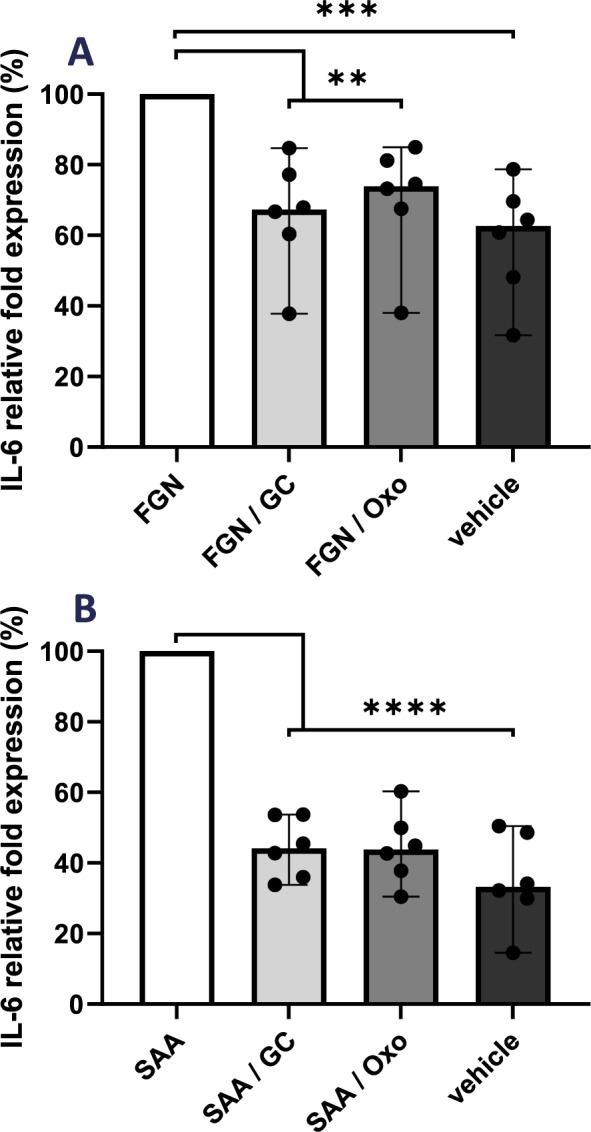
Effect of GC and TAK1 inhibition in IL-6 production in PBMCs. **A** Healthy volunteers´ PBMCs (*N* = 6) were pre-incubated with 5Z-7-oxozeanol (Oxo; 5 µM) or GC (10 µM) and stimulated for 24 h with fibrinogen (FGN; 0.10 mg/ml). The IL-6 production was measured by ELISA. **B** Same experiment as in A but PBMCs were stimulated with SAA (0.17 mg/ml). The graph-bars represent the median values. *p* < 0.01 (**); *p* < 0.001 (***); *p* < 0.0001 (****)

The previous experiments indicate that fibrinogen and SAA are antigens that appear increasingly expressed in C-GCA TABs. Moreover, it has been described that fibrinogen and SAA might bind TLR2 and TLR4 (Smiley 2001; Connolly et al. 2016). Thus, we assessed the anti-inflammatory effect of GCs and 5Z-7-oxozeanol (oxo) following TLR stimulation. Oxo targets transforming growth factor beta-activated kinase-1 (TAK1) (Wu et al. 2013), which is functional downstream of TLRs. Healthy volunteers´ PBMCs were pre-incubated with oxo or GCs, and they were stimulated with fibrinogen or SAA (Fig. 4A, B). The IL-6 production induced by fibrinogen (144.3 pg/ml) or SAA (110.3 pg/ml) was significantly reduced by oxo (to 62.3% and 42.0%) and by GCs (to 55.0% and 45.0%). These results indicate that the DAMPs fibrinogen and SAA induce IL-6 production, which is a main cytokine in GCA, and this inflammatory response may be decreased in vitro by TAK1-inhibition or GC treatment.

### Association of inflammatory markers in PMR and GCA

**Fig. 5:**
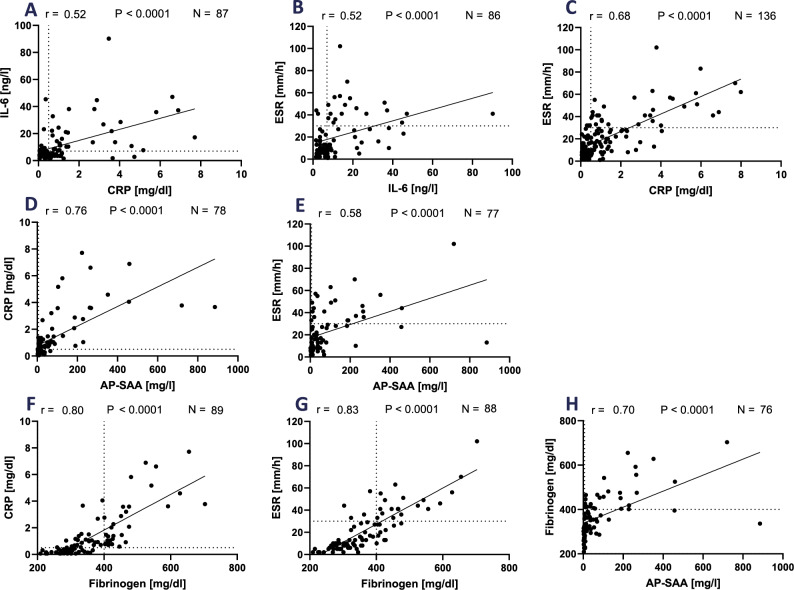
Association of IL-6, CRP, ESR, fibrinogen, and AP-SAA in patients with PMR (*N* = 139). **A-H** Data values were used for the association studies when both values were determined in the same blood sample. r: Spearman´s rank correlation coefficient; P: *p*-values, significant if *p* < 0.05 (*); *p* < 0.01(**); *p* < 0.001(***); *p* < 0.0001 (****). N: number of patients. IL-6: interleukin-6. CRP: C-reactive protein. ESR: erythrocyte sedimentation rate. AP-SAA: acute-phase serum amyloid A. Dotted line: reference values (indicated in Table S4)

Although GC treatment induces a strong decrease in ESR and CRP levels, after tapering, acute-phase proteins may remain elevated in some patients (Roche 1993, Salvarani 2003). The previous results point to a role of SAA and fibrinogen in the underlying inflammatory process in GCA. Thus, we set to analyse the association between the levels of standard inflammatory markers (e.g. IL-6, CRP, and ESR) and those of fibrinogen and SAA in a cohort of 139 PMR and 40 GCA patients (Table S2). The reference values for the different markers appeared in Table S4. As shown in Fig. 5A and B, in PMR patients´ plasma, IL-6 levels presented a low correlation coefficient with CRP (*r* = 0.52) and ESR (*r* = 0.52), respectively. The correlation coefficient between ESR and CRP was 0.68 (Fig. 5C). In contrast, the association between AP-SAA and CRP or ESR was relatively strong (*r* = 0.76 and 0.58, respectively; Fig. 5D, E). Remarkably, the highest association was observed when analysing fibrinogen and CRP (*r* = 0.80) or ESR (*r* = 0.83) (Fig. 5F, G). The new investigated parameters, fibrinogen and SAA, showed a relatively strong association (*r* = 0.70; Fig. 5H). The association of IL-6 with both of them was weaker (*r* = 0.61, *r* = 0.46; Fig. S6A, S6B).

**Fig. 6:**
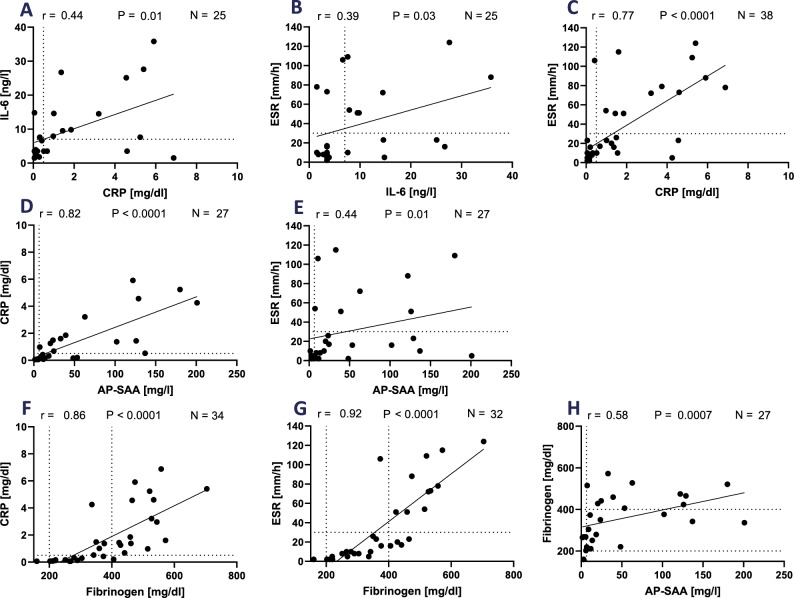
Association of IL-6, CRP, ESR, fibrinogen, and AP-SAA in patients with GCA (*N* = 40). **A-H** Data values were used for the association studies when both values were determined in the same blood sample. r: Spearman´s rank correlation coefficient; P: *p*-values significant if *p* < 0.05 (*); *p* < 0.01(**); *p* < 0.001(***); *p* < 0.0001 (****). N: number of patients. IL-6: interleukin-6. CRP: C-reactive protein. ESR: erythrocyte sedimentation rate. AP-SAA: acute-phase serum amyloid A. Dotted line: reference values (indicated in Table S4)

In the cohort of GCA patients´ plasma, we observed no association between IL-6 levels and CRP or ESR (*r* = 0.44 and 0.39, respectively; Fig. 6A, B). However, these two parameters associated strongly (*r* = 0.77; Fig. 6C). While AP-SAA levels robustly associated with CRP (*r* = 0.82; Fig. 6D), AP-SAA values associated under 0.50 with ESR (Fig. 6E). The levels of fibrinogen associated with CRP and ESR stronger than in the PMR patients (*r* = 0.86 and 0.92, respectively; Fig. 6F, G). Fibrinogen and AP-SAA associated positively also in these patients (*r* = 0.58; Fig. 6H), and IL-6 did not associate with both parameters (*r* = 0.34, *r* = 0.41; Fig. S6C, S6D).

## Discussion

This study is the first to show that at the time of diagnosis TLR2, TLR4, TLR7, and TLR8 are highly expressed in C-GCA samples and only expressed at low levels in TABs of controls. In addition, TABs from EC-GCA and PMR patients exhibited a slightly increased expression of TLR4. These results are highly relevant since we investigate the expression of inflammatory receptors (TLRs) and potential DAMPs in various stages within the spectrum of the disease: PMR, EC-GCA, and C-GCA.

Immune cells, such as macrophages infiltrating GCA-arteries, are predicted to express high levels of TLRs, as shown in the case of the co-staining of TLR8 with the macrophage marker CD68 in C-GCA TABs. However, other cell types (e.g. muscle cells –Fig. S7-; fibroblasts (Vink et al. 2002)) also seem to express TLRs, as we observed in control samples which are not populated by macrophages. This effect was more evident in C-GCA resident cells.

In response to endothelial injury signals, VSMCs migrate into the intima layer where they proliferate and produce pro-inflammatory cytokines (e.g. IL-6) (Piggott 2009). VSMCs express TLR2, TLR3, TLR4, and TLR9. While activation of TLR2, TLR3, and TLR4 leads to IL-6 production, activation of TLR7/8 and TLR9 induces the production of IL-12, TNF-α, IL1-ß and INF-α (Piggott 2009). We showed that TLR8 and TLR7 were highly expressed in C-GCA, but also other TLRs may play an important role, as GCA patients have high IL-6 plasma levels.

TLR8 and TLR7 recognise GU- and AU-rich sequences in ssRNA from viruses (e.g. influenza, coronaviruses) and also bacterial RNA (Mancuso et al. 2009). In this respect, a variety of infectious agents have been proposed as potential triggers of GCA development, although contradictory results have been reported (Ostrowski 2019). Thus, it is plausible that a viral infection might trigger the GCA inflammatory process, or alternatively, TLRs might be activated by endogenous ligands (O'Neill 2016), since some small interfering RNAs may ligate TLR7/8 (Hornung 2005).

We also observed increased TLR4 expression in C-GCA samples. TLR4 has been previously related to GCA since LPS treatment induces vasculitis in human TAs implanted into NOD-SCID mice (Ma-Krupa 2004). Additionally, the polymorphism rs4986790 (−896 A/G, Asp299Gly) in TLR4 was associated with GCA susceptibility in a Spanish cohort of biopsy-positive GCA patients (Palomino-Morales et al. 2009), although no association with ischemic symptoms was found in another study (Alvarez Rodriguez et al. 2011). DCs (which abundantly express TLRs) are localized at the arterial adventitia-media interface and play a key role in the initiation of GCA by polarizing T-cells (Weyand 2005). Hence, resident DCs may initiate a different inflammatory response depending on the TLR functionality and TLR-compensatory mechanisms. Thus, it would be interesting to compare TABs TLR expression and clinical response in patients with TLR polymorphisms.

Several TLRs are overexpressed at the site of inflammation in C-GCA TABs (between 2 and 4 times) in comparison to controls, yet the affinity of the TLR-antibodies for their epitopes is dissimilar. Thus, we cannot conclude which TLR was distinctively expressed at the highest level, which could give a hint of the alarmins involved in the inflammatory response. At present, the trigger of arterial inflammation (endogenous or exogenous) has not been unveiled. We described that the alarmins fibrinogen, HMGB-1, and SAA are overexpressed in TABs of GCA patients, and potentially might contribute to, or even initiate, the inflammatory process in GCA arteries. The increased fibrinogen expression is consistent with earlier studies that demonstrated the presence of fibrinogen and its degradation products in the serum of PMR and GCA patients, and that these levels decrease after starting GC therapy (Grau 1984; McCarthy et al. 2013). Also, HMGB-1 is a mediator of inflammation, as a ligand of TLR2, TLR4, and RAGE (Park et al. 2004; Tian et al. 2007), that can promote ECM degradation and activate cell autophagy (Fu 2020), and it has been involved in various autoimmune diseases (e.g. rheumatoid arthritis (Schierbeck et al. 2013)). Endothelial cells and activated immune cells (e.g. macrophages, DCs) may secrete HMGB-1 that, in turn stimulates DCs, leading to T-cell polarization (Dumitriu et al. 2005). We found marked expression of HMGB-1 in the media of PMR, EC-GCA, and C-GCA TABs in comparison to controls, yet it is uncertain whether HMGB-1 is the inflammation-triggering DAMP, or its expression increases following cell death after the inflammation has settled. Noteworthy, anti-HMGB-1 neutralizing antibodies show protective effects in experimental models of inflammation. Thus, anti-HMGB-1 based therapies may also be useful in chronic inflammatory diseases (Wang et al. 2015).

SAA exhibits high affinity for HDL (high-density lipoprotein). However, during the acute-phase response, the levels of lipid-free SAA in serum appear increased. Circulating SAA is elevated in GCA patients in comparison to controls, and it has been proposed as a regulator of TA inflammation, angiogenesis, and invasion (O'Neill et al. 2015). Primarily, SAA is produced by the liver, although extra-hepatic expression has been described for macrophages and VSMCs (Eklund 2012). SAA can activate the monocyte chemoattractant protein-1 (MCP-1) in VSMCs in a TLR2- and TLR4-dependent way (Schuchardt et al. 2019). In this line, we found increased expression of SAA in C-GCA TABs, where it might amplify the stimulation of macrophages via TLR2, as it does in the ex vivo model of TAs (O'Neill et al. 2015). SAA expression in EC-GCA was also significantly increased, although EC-GCA TABs were negative. Thus, it might be an indicator of the disease regardless of the predominant phenotype.

P-glycoprotein expression and function can be modulated by TLR activity, and GC-resistance in patients might be due to overexpression of p-glycoprotein (Smutny 2019; Frank et al. 2015). Accordingly, we found P-glycoprotein significantly overexpressed in the intima and media lamina, and at the intermediate border of TABs from C-GCA patients. It would be interesting to assess whether higher P-glycoprotein expression profiles in TABs correlate with GC-resistances or flares, in order to timely manage those patients with alternative therapies.

From the pathologists´ perspective, if our studies would be further validated by other groups with a larger sample size, staining and IF analysis of SAA, HMGB-1, fibrinogen, and TLRs in C-GCA TABs might contribute to both the diagnosis and classification of the pathology status. Particularly, in those patients with concurrent pathologies or when the histological evaluation becomes challenging due to loss artery integrity (for example resulting from artery-sample slicing).

In addition, the assessment of early antigen expression in PMR and EC-GCA samples could help us to better understand the intricate spectrum of GCA phenotypes, although at this point, we cannot conclude whether the observed TLR-staining pattern is the cause or the consequence of disease progression from PMR to GCA. In order to assess the evolution of arterial marker expression with the course of the disease, it would be necessary to analyse TABs at the start and at the end of the GC therapy, what is not ethically feasible. Considering the increased expression of innate immunity mediators in TABs, and the inhibitory effect on IL-6 production exerted by GC and 5Z-7-oxozeanol in fibrinogen- or SAA-treated immune cells, we speculate about the potential therapeutic effect of TLR antagonists (such as hydroxychloroquine for TLR7) to reduce the inflammation in GCA. To this respect, the results of one randomized control study by Sailler et al. (2009) published in abstract form, showed no evidence of efficacy of hydroxychloroquine when compared to GCs.

We have observed a strong association between SAA levels and the inflammatory markers ESR and CRP in the plasma of PMR and GCA patients. This association was even stronger for fibrinogen. Plasma fibrinogen levels and inflammatory markers correlation has been observed in previous studies (Grau 1984, McCarthy et al. 2014). Yet, both fibrinogen and SAA are not considered as typical biomarkers to assess GCA or PMR disease activity and progression. It is uncertain whether the levels detected in plasma are derived from the vascular remodelling of the affected arteries or whether the observed expression in the arteries is due to deposition of the circulating antigen.

We are aware of some limitations of this study. First, the sample size of the TABs studied is a critical consideration. Yet, despite the small sample size, the IF staining showed consistent results in the range of samples analysed. Secondly, although antibody specificity remains a concern in tissue sample staining, in instances where the specificity was an issue (e.g. CD68, SAA, and TLR2), we conducted screenings using different antibodies. Thirdly, our results indicate increased expression of TLRs in CD68^+^ macrophages and, as presumed from tissue location, morphology and smooth muscle actin (SMA) staining (Fig. S7), in myocytes. However, other cell types (e.g. lymphocytes) were not examined. Further, the association studies are limited by the heterogeneous group of patients included, due to the retrospective character of the study. Some patients were under GC treatment at the time of the blood-sampling, what can influence the levels of serum markers, as previous studies have uncovered. GC treatment leads to fast (within hours) decline in plasma IL-6, ESR, and CRP levels, which correlates with the remission of clinical symptoms. However, GC therapy does not correct the underlying mechanism that leads to IL-6 production (fast increase of plasma IL-6 after short-term withdrawal of GCs) as acute-phase proteins remain slightly elevated above normal levels (Roche 1993; Park 1981; Cid et al. 1996). A proper stratification according to disease status, received medication, and baseline levels of fibrinogen and SAA in plasma might be focus of a prospective study in order to increase the accuracy to designate fibrinogen and SAA as potential disease biomarkers, which was not the purpose of this study.

## Conclusion

In conclusion, the innate immune receptors TLR2, TLR4, TLR7, TLR8, and potential alarmins (i.e. HMGB-1, SAA, fibrinogen) are highly expressed in TABs of C-GCA patients, and the first two alarmins are also expressed in EC-GCA. Additionally, p-glycoprotein expression appeared increased in C-GCA samples. Future studies with a larger cohort of patients might confirm whether the here proposed TLRs and alarmins might be used as validation tools for the diagnosis of TABs if further confirmed. Moreover, the levels of SAA and fibrinogen in patients´ plasma associate with the standard inflammatory parameters ESR and CRP better than IL-6 does, and thus, they might be more relevant for the laboratory monitoring of this condition as currently expected, especially in patients under treatment with IL-6R antagonists.

## Supplementary Information

Supplementary Material 1: Table S1: Clinical features of patients included in the immunofluorescence study. Table S2: Description of patients whose data sets are included in the association analyses. Table S3: Antibodies used in the IF studies. Table S4: Reference values of clinical inflammatory markers. Figure S1: Scheme used for the antigen quantification in IF images of TABs. Figure S2: TABs staining with the secondary antibodies used in the IF in patients with C-GCA. Figure S3: TABs staining with isotype-control antibodies in controls and patients with C-GCA. Figure S4: Screening of TLR2, SAA-1, and CD68 antibodies. Figure S5: Staining of HMGB-1 and Hoechst of controls, PMR, and EC-GCA patients. Figure S6: Association of IL-6 with Fibrinogen or AP-SAA in PMR and GCA patients. Figure S7: Expression of TLR8 and SMA in TABs of control and GCA samples. Figure S8: TLR8 expression detected with two different anti-TLR8 antibodies.

## Data Availability

The data generated during and/or analysed during the current study are available from the corresponding author on reasonable request.

## Abbreviations

CRP: C-reactive protein
DCs: Dendritic cells
ECM: Extracellular matrix
EEM: External elastic membrane
ESR: Erythrocyte sedimentation rate
FGN: Fibrinogen
GCA: Giant cell arteritis
HMGB-1: High mobility group box-1
IEM: Internal elastic membrane
IF: Immunofluorescence
GC: Glucocorticoids
MUI: Medical University of Innsbruck
PBMCs: Peripheral blood mononuclear cells
PMR: Polymyalgia rheumatica
SAA: Serum amyloid A
SMA: Smooth muscle actin
TA: Temporal artery
TAB: Temporal artery biopsy
TAK1: Transforming growth factor- beta-activated kinase 1
TLR: Toll-like receptor
UHLP: University Hospital La Princesa
VSCMs: Vascular smooth muscle cells

## References

[CR1] Alba MA, Garcia-Martinez A, Prieto-Gonzalez S, Tavera-Bahillo I, Corbera-Bellalta M, Planas-Rigol E, et al. Relapses in patients with giant cell arteritis: prevalence, characteristics, and associated clinical findings in a longitudinally followed cohort of 106 patients. Medicine (Baltimore). 2014;93(5):194–201. 25181312 10.1097/MD.0000000000000033 PMC4602452

[CR2] Alvarez Rodriguez L, Lopez-Hoyos M, Mata C, Fontalba A, Calvo Alen J, Marin MJ, et al. Expression and function of toll-like receptors in peripheral blood mononuclear cells of patients with polymyalgia rheumatica and giant cell arteritis. Ann Rheum Dis. 2011;70(9):1677–83. 21670089 10.1136/ard.2010.140194

[CR3] Baldini M, Maugeri N, Ramirez GA, Giacomassi C, Castiglioni A, Prieto-Gonzalez S, et al. Selective up-regulation of the soluble pattern-recognition receptor pentraxin 3 and of vascular endothelial growth factor in giant cell arteritis: relevance for recent optic nerve ischemia. Arthritis Rheum. 2012;64(3):854–65. 21989653 10.1002/art.33411

[CR4] Castaneda S, Prieto-Pena D, Vicente-Rabaneda EF, Triguero-Martinez A, Roy-Vallejo E, Atienza-Mateo B, et al. Advances in the treatment of giant cell arteritis. J Clin Med. 2022;11(6):1588. 35329914 10.3390/jcm11061588 PMC8954453

[CR5] Ciccia F, Ferrante A, Guggino G, Cavazza A, Salvarani C, Rizzo A. CD3 immunohistochemistry is helpful in the diagnosis of giant cell arteritis. Rheumatology (Oxford). 2018;57(8):1377–80. 29697809 10.1093/rheumatology/key106

[CR6] Cid MC, Monteagudo J, Oristrell J, Vilaseca J, Pallares L, Cervera R, et al. Von Willebrand factor in the outcome of temporal arteritis. Ann Rheum Dis. 1996;55(12):927–30. 9014589 10.1136/ard.55.12.927 PMC1010347

[CR7] Connolly M, Rooney PR, McGarry T, Maratha AX, McCormick J, Miggin SM, et al. Acute serum amyloid A is an endogenous TLR2 ligand that mediates inflammatory and angiogenic mechanisms. Ann Rheum Dis. 2016;75(7):1392–8. 26290589 10.1136/annrheumdis-2015-207655

[CR8] Dasgupta B, Cimmino MA, Kremers HM, Schmidt WA, Schirmer M, Salvarani C, et al. 2012 Provisional classification criteria for polymyalgia rheumatica: a European League Against Rheumatism/American College of Rheumatology collaborative initiative. Arthritis Rheum. 2012;64(4):943–54. 22389040 10.1002/art.34356

[CR9] Dejaco C, Duftner C, Buttgereit F, Matteson EL, Dasgupta B. The spectrum of giant cell arteritis and polymyalgia rheumatica: revisiting the concept of the disease. Rheumatology (Oxford). 2017;56(4):506–15. 27481272 10.1093/rheumatology/kew273

[CR10] Deng J, Younge BR, Olshen RA, Goronzy JJ, Weyand CM. Th17 and Th1 T-cell responses in giant cell arteritis. Circulation. 2010;121(7):906–15. 20142449 10.1161/CIRCULATIONAHA.109.872903 PMC2837465

[CR11] Dumitriu IE, Baruah P, Valentinis B, Voll RE, Herrmann M, Nawroth PP, et al. Release of high mobility group box 1 by dendritic cells controls T cell activation via the receptor for advanced glycation end products. J Immunol. 2005;174(12):7506–15. 15944249 10.4049/jimmunol.174.12.7506

[CR12] Eklund KK, Niemi K, Kovanen PT. Immune functions of serum amyloid A. Crit Rev Immunol. 2012;32(4):335–48. 23237509 10.1615/critrevimmunol.v32.i4.40

[CR13] Ferrigno I, Bonacini M, Rossi A, Nicastro M, Muratore F, Boiardi L, et al. Genes deregulated in giant cell arteritis by Nanostring nCounter gene expression profiling in temporal artery biopsies. RMD Open. 2024. 10.1136/rmdopen-2024-004600. 39317454 10.1136/rmdopen-2024-004600 PMC11423731

[CR14] Frank M, Hennenberg EM, Eyking A, Runzi M, Gerken G, Scott P, et al. Tlr signaling modulates side effects of anticancer therapy in the small intestine. J Immunol. 2015;194(4):1983–95. 25589072 10.4049/jimmunol.1402481 PMC4338614

[CR15] Fu B, Lu X, Zhao EY, Wang JX, Peng SM. HMGB1-induced autophagy promotes extracellular matrix degradation leading to intervertebral disc degeneration. Int J Clin Exp Pathol. 2020;13(9):2240–8. 33042328 PMC7539879

[CR16] Gonzalez-Gay MA. Giant cell arteritis and polymyalgia rheumatica: two different but often overlapping conditions. Semin Arthritis Rheum. 2004;33(5):289–93. 15079759 10.1016/j.semarthrit.2003.09.007

[CR17] Gonzalez-Gay MA, Castaneda S, Llorca J. Giant cell arteritis: visual loss is our major concern. J Rheumatol. 2016;43(8):1458–61. 27481989 10.3899/jrheum.160466

[CR18] Gonzalez-Gay MA, Matteson EL, Castaneda S. Polymyalgia rheumatica. Lancet. 2017;390(10103):1700–12. 28774422 10.1016/S0140-6736(17)31825-1

[CR19] Gonzalez-Gay MA, Prieto-Pena D, Martinez-Rodriguez I, Calderon-Goercke M, Banzo I, Blanco R, et al. Early large vessel systemic vasculitis in adults. Best Pract Res Clin Rheumatol. 2019;33(4):101424. 31810548 10.1016/j.berh.2019.06.006

[CR20] Gonzalez-Gay MA, Vicente-Rabaneda EF, Heras-Recuero E, Castaneda S. Polymyalgia rheumatica: when should we suspect an underlying large vessel vasculitis? Clin Exp Rheumatol. 2023;41(4):774–6. 36995322 10.55563/clinexprheumatol/3bozph

[CR21] Grau RG, Kassan SS, Franks JJ, Kaplan H, Walker SH, Tan EM. Fibrin(ogen)olysis in polymyalgia rheumatica and temporal arteritis: preliminary findings on association with disease activity. Ann Rheum Dis. 1984;43(5):721–4. 6497463 10.1136/ard.43.5.721 PMC1001515

[CR22] Hayreh SS, Podhajsky PA, Zimmerman B. Ocular manifestations of giant cell arteritis. Am J Ophthalmol. 1998;125(4):509–20. 9559737 10.1016/s0002-9394(99)80192-5

[CR23] Hornung V, Guenthner-Biller M, Bourquin C, Ablasser A, Schlee M, Uematsu S, et al. Sequence-specific potent induction of IFN-alpha by short interfering RNA in plasmacytoid dendritic cells through TLR7. Nat Med. 2005;11(3):263–70. 15723075 10.1038/nm1191

[CR24] Hunder GG, Bloch DA, Michel BA, Stevens MB, Arend WP, Calabrese LH, et al. The American college of rheumatology 1990 criteria for the classification of giant cell arteritis. Arthritis Rheum. 1990;33(8):1122–8. 2202311 10.1002/art.1780330810

[CR25] Ma-Krupa W, Jeon MS, Spoerl S, Tedder TF, Goronzy JJ, Weyand CM. Activation of arterial wall dendritic cells and breakdown of self-tolerance in giant cell arteritis. J Exp Med. 2004;199(2):173–83. 14734523 10.1084/jem.20030850 PMC2211768

[CR26] Mancuso G, Gambuzza M, Midiri A, Biondo C, Papasergi S, Akira S, et al. Bacterial recognition by TLR7 in the lysosomes of conventional dendritic cells. Nat Immunol. 2009;10(6):587–94. 19430477 10.1038/ni.1733

[CR27] McCarthy EM, MacMullan PA, Al-Mudhaffer S, Madigan A, Donnelly S, McCarthy CJ, et al. Plasma fibrinogen is an accurate marker of disease activity in patients with polymyalgia rheumatica. Rheumatology (Oxford). 2013;52(3):465–71. 23125391 10.1093/rheumatology/kes294

[CR28] McCarthy EM, MacMullan PA, Al-Mudhaffer S, Madigan A, Donnelly S, McCarthy CJ, et al. Plasma fibrinogen along with patient-reported outcome measures enhances management of polymyalgia rheumatica: a prospective study. J Rheumatol. 2014;41(5):931–7. 24692520 10.3899/jrheum.131055

[CR29] Muniz Castro HM, Bhattacharjee MB, Chaudhry IA, Chuang AZ, Mankiewicz KA, Adesina OO. Diagnosis of giant cell arteritis using clinical, laboratory, and histopathological findings in patients undergoing temporal artery biopsy. Clin Neurol Neurosurg. 2022;221:107377. 35932586 10.1016/j.clineuro.2022.107377

[CR30] O’Neill L, Molloy ES. The role of toll like receptors in giant cell arteritis. Rheumatology (Oxford). 2016;55(11):1921–31. 26893518 10.1093/rheumatology/kew001

[CR31] O’Neill L, Rooney P, Molloy D, Connolly M, McCormick J, McCarthy G, et al. Regulation of inflammation and angiogenesis in giant cell arteritis by acute-phase serum amyloid A. Arthritis Rheumatol. 2015;67(9):2447–56. 26016600 10.1002/art.39217

[CR32] Ostrowski RA, Metgud S, Tehrani R, Jay WM. Varicella zoster virus in giant cell arteritis: a review of current medical literature. Neuroophthalmology. 2019;43(3):159–70. 31312240 10.1080/01658107.2019.1604763 PMC6620003

[CR33] Palomino-Morales R, Torres O, Vazquez-Rodriguez TR, Morado IC, Castaneda S, Callejas-Rubio JL, et al. Association between toll-like receptor 4 gene polymorphism and biopsy-proven giant cell arteritis. J Rheumatol. 2009;36(7):1501–6. 19531762 10.3899/jrheum.081286

[CR34] Park JR, Jones JG, Hazleman BL. Relationship of the erythrocyte sedimentation rate to acute phase proteins in polymyalgia rheumatica and giant cell arteritis. Ann Rheum Dis. 1981;40(5):493–5. 6171213 10.1136/ard.40.5.493 PMC1000787

[CR35] Park JS, Svetkauskaite D, He Q, Kim JY, Strassheim D, Ishizaka A, et al. Involvement of toll-like receptors 2 and 4 in cellular activation by high mobility group box 1 protein. J Biol Chem. 2004;279(9):7370–7. 14660645 10.1074/jbc.M306793200

[CR36] Piggott K, Biousse V, Newman NJ, Goronzy JJ, Weyand CM. Vascular damage in giant cell arteritis. Autoimmunity. 2009;42(7):596–604. 19657775 10.1080/08916930903002495 PMC4271842

[CR37] Ponte C, Grayson PC, Robson JC, Suppiah R, Gribbons KB, Judge A, et al. 2022 American College of Rheumatology/EULAR classification criteria for giant cell arteritis. Ann Rheum Dis. 2022a;81(12):1647–53. 36351706 10.1136/ard-2022-223480

[CR38] Ponte C, Grayson PC, Robson JC, Suppiah R, Gribbons KB, Judge A, et al. 2022 American College of Rheumatology/EULAR Classification Criteria for Giant Cell Arteritis. Arthritis Rheumatol. 2022b;74(12):1881–9. 36350123 10.1002/art.42325

[CR39] Pryshchep O, Ma-Krupa W, Younge BR, Goronzy JJ, Weyand CM. Vessel-specific toll-like receptor profiles in human medium and large arteries. Circulation. 2008;118(12):1276–84. 18765390 10.1161/CIRCULATIONAHA.108.789172 PMC2748975

[CR40] Roche NE, Fulbright JW, Wagner AD, Hunder GG, Goronzy JJ, Weyand CM. Correlation of interleukin-6 production and disease activity in polymyalgia rheumatica and giant cell arteritis. Arthritis Rheum. 1993;36(9):1286–94. 8216422 10.1002/art.1780360913

[CR41] Sailler L, Lapeyre-Mestre M, Geffray L, Letellier P, Liozon E, De La Roque P, et al. Adding hydroxychloroquine to prednisone does not improve the outcome in giant cell arteritis: a double blind randomized controlled trial. Arthritis Rheum. 2009;60(Suppl 10):1972.

[CR42] Salvarani C, Cantini F, Boiardi L, Hunder GG. Laboratory investigations useful in giant cell arteritis and Takayasu’s arteritis. Clin Exp Rheumatol. 2003;21(6 Suppl 32):S23–8. 14740424

[CR43] Samson M, Dasgupta B, Sammel AM, Salvarani C, Pagnoux C, Hajj-Ali R, et al. Targeting interleukin-6 pathways in giant cell arteritis management: A narrative review of evidence. Autoimmun Rev. 2024:103716.10.1016/j.autrev.2024.103716 10.1016/j.autrev.2024.103716 39644981

[CR44] Schierbeck H, Pullerits R, Pruunsild C, Fischer M, Holzinger D, Laestadius A, et al. HMGB1 levels are increased in patients with juvenile idiopathic arthritis, correlate with early onset of disease, and are independent of disease duration. J Rheumatol. 2013;40(9):1604–13. 23858044 10.3899/jrheum.120987

[CR45] Schuchardt M, Prufer N, Tu Y, Herrmann J, Hu XP, Chebli S, et al. Dysfunctional high-density lipoprotein activates toll-like receptors via serum amyloid a in vascular smooth muscle cells. Sci Rep. 2019;9(1):3421. 30833653 10.1038/s41598-019-39846-3 PMC6399289

[CR46] Slobodin G, Lurie M, Bejar J, Rozenbaum M, Boulman N, Rosner I. Biopsy-negative giant cell arteritis: does anti-CD83 immunohistochemistry advance the diagnosis? Eur J Intern Med. 2007;18(5):405–8. 17693229 10.1016/j.ejim.2007.01.005

[CR47] Smiley ST, King JA, Hancock WW. Fibrinogen stimulates macrophage chemokine secretion through toll-like receptor 4. J Immunol. 2001;167(5):2887–94. 11509636 10.4049/jimmunol.167.5.2887

[CR48] Smutny T, Barvik I, Veleta T, Pavek P, Soukup T. Genetic predispositions of glucocorticoid resistance and therapeutic outcomes in polymyalgia rheumatica and giant cell arteritis. J Clin Med. 2019;8(5):582. 31035618 10.3390/jcm8050582 PMC6572549

[CR49] Tian J, Avalos AM, Mao SY, Chen B, Senthil K, Wu H, et al. Toll-like receptor 9-dependent activation by DNA-containing immune complexes is mediated by HMGB1 and RAGE. Nat Immunol. 2007;8(5):487–96. 17417641 10.1038/ni1457

[CR50] Tomelleri A, van der Geest KSM, Khurshid MA, Sebastian A, Coath F, Robbins D, et al. Disease stratification in GCA and PMR: state of the art and future perspectives. Nat Rev Rheumatol. 2023;19(7):446–59. 37308659 10.1038/s41584-023-00976-8

[CR51] Ugrinova I, Pashev IG, Pasheva EA. Nucleosome binding properties and co-remodeling activities of native and in vivo acetylated HMGB-1 and HMGB-2 proteins. Biochemistry. 2009;48(27):6502–7. 19522541 10.1021/bi9004304

[CR52] Vink A, Schoneveld AH, van der Meer JJ, van Middelaar BJ, Sluijter JP, Smeets MB, et al. In vivo evidence for a role of toll-like receptor 4 in the development of intimal lesions. Circulation. 2002;106(15):1985–90. 12370224 10.1161/01.cir.0000032146.75113.ee

[CR53] Wang C, de Souza AW, Westra J, Bijl M, Chen M, Zhao MH, et al. Emerging role of high mobility group box 1 in ANCA-associated vasculitis. Autoimmun Rev. 2015;14(11):1057–65. 26209906 10.1016/j.autrev.2015.07.010

[CR54] Weyand CM, Goronzy JJ. Immunology of giant cell arteritis. Circ Res. 2023;132(2):238–50. 36656970 10.1161/CIRCRESAHA.122.322128 PMC9870348

[CR55] Weyand CM, Ma-Krupa W, Pryshchep O, Groschel S, Bernardino R, Goronzy JJ. Vascular dendritic cells in giant cell arteritis. Ann N Y Acad Sci. 2005;1062:195–208. 16461802 10.1196/annals.1358.023

[CR56] Wu J, Powell F, Larsen NA, Lai Z, Byth KF, Read J, et al. Mechanism and in vitro pharmacology of TAK1 inhibition by (5Z)-7-Oxozeaenol. ACS Chem Biol. 2013;8(3):643–50. 23272696 10.1021/cb3005897

