# Supplementary material for: Increased expression of Toll-like receptors and associated alarmins in temporal arteries of patients with giant cell arteritis
Source: Mol Med. 2025 Nov 12;31:331. doi: 10.1186/s10020-025-01390-4 (PMC12613359; doi:10.1186/s10020-025-01390-4)
Supplement: Supplementary file 1 — Supplementary Material 1: Table S1: Clinical features of patients included in the immunofluorescence study. Table S2: Description of patients whose data sets are included in the association analyses. Table S3: Antibodies used in the IF studies. Table S4: Reference values of clinical inflammatory markers. Figure S1: Scheme used for the antigen quantification in IF images of TABs. Figure S2: TABs staining with the secondary antibodies used in the IF in patients with C-GCA. Figure S3: TABs staining with isotype-control antibodies in controls and patients with C-GCA. Figure S4: Screening of TLR2, SAA-1, and CD68 antibodies. Figure S5: Staining of HMGB-1 and Hoechst of controls, PMR, and EC-GCA patients. Figure S6: Association of IL-6 with Fibrinogen or AP-SAA in PMR and GCA patients. Figure S7: Expression of TLR8 and SMA in TABs of control and GCA samples. Figure S8: TLR8 expression detected with two different anti-TLR8 antibodies. [file 10020_2025_1390_MOESM1_ESM.pdf]

Increased expression of Toll-like receptors and associated alarmins in temporal arteries of patients with giant cell arteritis

Seidlberger S, Schirmer M, Wietzorrek G, Jiménez-Heffernan JA, Pardines M, de las Fuentes Monreal M, González-Gay MA, Castañeda S, Santos-Sierra S.

Table S1: Clinical features of patients included in the immunofluorescence study.

Table S2: Description of patients whose data sets are included in the correlation analyses.

Table S3: Antibodies used in the IF studies.

Table S4: Reference values of clinical inflammatory markers.

Figure S1: Scheme used for the antigen quantification in IF images of TABs.

Figure S2: TABs staining with the secondary antibodies used in the IF in patients with C-GCA.

Figure S3: TABs staining with isotype-control antibodies in controls and patients with C-GCA.

Figure S4: Screening of TLR2, SAA-1, and CD68 antibodies.

Figure S5: Staining of HMGB-1 and Hoechst of controls, PMR, and EC-GCA patients.

Figure S6: Association of IL-6 with Fibrinogen or AP-SAA in PMR and GCA patients.

Figure S7: Expression of TLR8 and SMA in TABs of control and GCA samples.

Figure S8: TLR8 expression detected with two different anti-TLR8 antibodies.

ID.	Age	Sex	Class	Diagnosis	Biopsy	Case history	Medication at the time of biopsy	Inflammatory blood markers at the time of biopsy	Type of imaging
**1**	87	F	C	Inflammatory disease of unknown origin. Following monitoring, GCA ruled out according to EULAR criteria	Negative	Systemic inflammation + visual loss	Nothing	ESR 51; CRP 3.65 mg/dL	PET negative, but done 3 months after starting therapy with GC
**2**	86	M	C	Acute inflammatory disease of unknown origin	Negative	Clinical picture suggestive of PMR (systemic response)	Prednisone 15 mg/d x 10 days	ESR 47; CRP 3.47 mg/dL	PET normal or negative two months after starting GC
**3**	60	F	C	Polyarthrosis/osteoarthritis	Negative	Inflammatory myalgia (systemic response +/-)	Prednisone 10 mg/d x 4 months	ESR 9; CRP 0.41 mg/dL	None
**4**	67	M	C	Rheumatoid arthritis	Negative	Systemic inflammatory picture	Prednisone 15 mg/d x 2 days	ESR 50; CRP 0.94 mg/dL	None
**5**	87	M	C	Immunomediated necrotizing myositis	Negative	Systemic inflammatory picture + recent headache	RTX + IVIG + MTX 12.5 mg/week	ESR 120; CRP 0.38 mg/dL	None
**6**	92	F	C	No diagnosis; severe anaemia, exitus	Negative	Constitutional syndrome (systemic response)	Nothing	ESR 107; CRP 7.13 mg/dL	None
**7**	85	F	C	Myalgia + Recent onset headache. No systemic picture	Negative	Absence of an inflammatory systemic response	Nothing	ESR 6; CRP 0.10 mg/dL	None
**8**	74	F	PMR		Negative		Prednisone 20 mg/d x 7 days	ESR 64; CRP 4 mg/dL	None
**9**	75	F	PMR		Negative		Prednisone 1.25 mg/d x 1 month	ESR 98; CRP 3.47 mg/dL	PET: Sub deltoid bursitis in both shoulders typical of PMR
**10**	82	M	PMR		Negative		Nothing	ESR 42; CRP 1.62 mg/dL	Thoracoabdominal CT normal
**11**	68	F	PMR		Negative		Prednisone 30 mg/d x 7 days	ESR 91; CRP 4.64 mg/dL	PET: negative, but done 6 months after starting therapy with GC
**12**	88	M	PMR		Negative		MTX 15 mg/week	ESR 36; CRP 0.12 mg/dL	None
**13**	77	M	EC-GCA		Negative		Prednisone 40 mg/d x 10 days	ESR 48; CRP 1.12 mg/dL	PET negative, but done 9 months after starting therapy with GC
**14**	75	F	EC-GCA		Negative		Nothing	ESR ND; CRP 11.85 mg/dL	None
**15**	79	F	EC-GCA		Negative		MPS 500 mg/iv x 3 days	ESR 46; CRP 0.58 mg/dL	PET negative, but done 18 months after starting therapy with GC
**16**	69	F	EC-GCA		Negative		MPS 500 mg/iv only 1 day	ESR 70; CRP 10.73 mg/dL	PET negative, but done 5 months after starting therapy with GC
**17**	86	F	EC-GCA		Negative		Prednisone 60 mg/d x 5 days	ESR 82; CRP 4.50 mg/dL	None
**18**	64	F	EC-GCA		Negative		MPS 125 mg/d x 3 days	ESR 116; CRP 11.29 mg/dL	PET normal/negative
**19**	72	M	C-GCA		Positive		Prednisone 20 mg/d x 15 days	ESR 94; CRP 13.69 mg/dL	PET/CT performed 2 months after diagnosis showing hypercaptation only in left temporal artery
**20**	89	M	C-GCA		Positive		MPS 250 mg/iv only 1 day	ESR 28; CRP 15.38 mg/dL	Aortic & supra-aortic CT PET negative/normal
**21**	79	F	C-GCA		Positive		Nothing	ESR 92; CRP 8.16 mg/dL	PET Negative/normal
**22**	86	F	C-GCA		Positive		Prednisone 60 mg/d x 3 days	ESR 22; CRP 0.78 mg/dL	None
**23**	81	F	C-GCA		Positive		Nothing	ESR 89; CRP 1.63 mg/dL	PET/CT performed 2 months after TAB negative
**24**	77	F	C-GCA		Positive		Prednisone 50 mg/d x 2 days	ESR 52; CRP 1.59 mg/dL	PET Periarticular hypercaptation in shoulders and spine, two years later

Table S1: Clinical features of patients included in the immunofluorescence study. ID: identifier; M: male; F: female; C-GCA: cranial giant cell arteritis; EC-GCA: extra-cranial giant cell arteritis; PMR: polymyalgia rheumatica; C: control; TAB: temporal artery biopsy. CRP: C-reactive protein; ESR: Erythrocyte sedimentation rate; IVIG: Intravenous immunoglobulins; MPS: Methylprednisolone intravenous boluses; MTX: Methotrexate; RTX: Rituximab.

		PMR	GCA
**Sex**	**Total**	139	40
	**Female**	77	25
	**Male**	62	15
**Age**	**Total**	53-98 (median 73)	55-87 (median 73)
	**Female**	53-84 (median 72)	55-85 (median 70)
	**Male**	53-98 (median 73.5)	60-87 (median 76)
**GC**		78	14
**MTX**		4	2
**TCZ**			4
**GC/MTX**		15	10
**GC/TCZ**		2	3

Table S2: Description of patients whose data sets are included in the correlation analyses (N=179).
Patients with missing laboratory values were not included in the corresponding correlation analysis. Patients treated with TCZ were not included in the IL-6 correlation analysis.
GC: Glucocorticoids; MTX: Methotrexate; TCZ: Tocilizumab; PMR: Polymyalgia rheumatica; GCA: Giant cell arteritis.

Epitope	Species reactivity	Host	Company	Identifier
Fibrinogen	Human	Rabbit	Sigma/Merck	#HPA001900
HMGB1	Human	Mouse	Sigma/Merck	#WH0003146M8
SAA1	Human	Mouse	Sigma/Merck	#AMAB91543
SAA2	Human	Rabbit	ThermoFisher	#13192-1-AP
U1C2 (MDR1 P-glycoprotein)	Human	Mouse	Millipore	#MAB4334Z
SMA	Human	Mouse	Sigma/Merck	#ZMS1004
TLR2 (1J19)	Human	Rabbit	Sigma/Merck	#ZRB2232
TLR2	Human	Rabbit	Cell Signaling	#2229
TLR2	Human	Rat	InvivoGen	#pab-hsttlr2
TLR4	Human	Mouse	Sigma/Merck	#SAB1404475
TLR7	Human	Rabbit	Sigma/Merck	#PRS3269
TLR8	Human	Rabbit	Sigma/Merck	#HPA001608
TLR8	Human	Rabbit	Sigma/Merck	#SAB3500307
CD 68 (D4B9C)	Human	Rabbit	CellSignalingTechnology	#76437
CD 68	Human	Rabbit	Sigma/Merck	#ZRB1427
CD 68	Human	Mouse	Sigma/Merck	#AMAB90873
CD 68	Human	Mouse	Sigma/Merck	#AMAB90874
CD83	Human	Rabbit	Sigma/Merck	#ZRB2993
IgG2a E5Y6Q	Human	Mouse	CellSignalingTechnology	#61656
IgG2b E7Q5L	Human	Mouse	CellSignalingTechnology	#53484
IgG Normal Rabbit	Human	Rabbit	Sigma/Merck	#NI01
Alexa Fluor™ 488	Rabbit	Goat	InvitroGen	#A-11034
Alexa Fluor™ 594	Mouse	Goat	InvitroGen	#A-11032
Alexa Fluor™ 594	Rat	Goat	InvitroGen	#A-11007
CF™350	Rabbit	Goat	Sigma/Merck	#SAB4600015

Table S3. Antibodies used in the IF studies.

Variable		Reference value	Units
**C-reactive protein**	CRP	0.0 – 0.5	mg/dl
**Interleukin-6**	IL-6	0.0 – 7.0	ng/l
**Acute phase-Serum Amyloid A**	AP-SAA	0.0 – 6.4	mg/l
**Fibrinogen**		200 – 400	mg/dl
**Erythrocyte sedimentation rate**	ESR	0 - 30	mm/hour

Table S4: Reference values of clinical inflammatory markers .

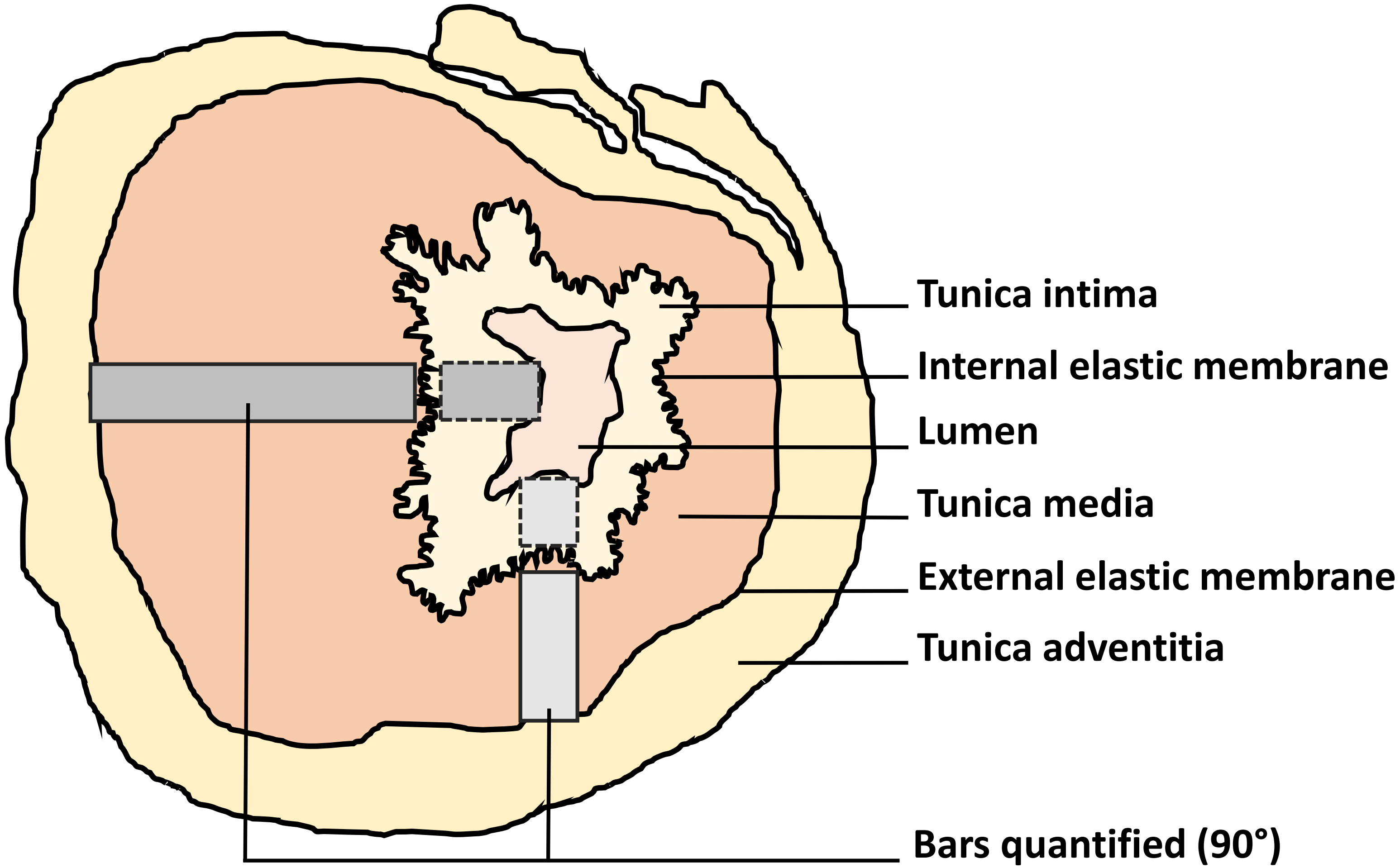

Figure S1: Measurement of the immunofluorescence intensity at the site of inflammation, or broader site in the artery (dark gray color), and 90° degrees (ligh gray color) displaced from the first bar area (width, 65 µm). The mean intensity of both areas was calculated (Image J). The adventitia was left out of the quantification due to the high background staining in all samples. Additionally, for those antibodies that showed high background staining of the elastic membrane, only the tunica media was measured (not to obscure the results).

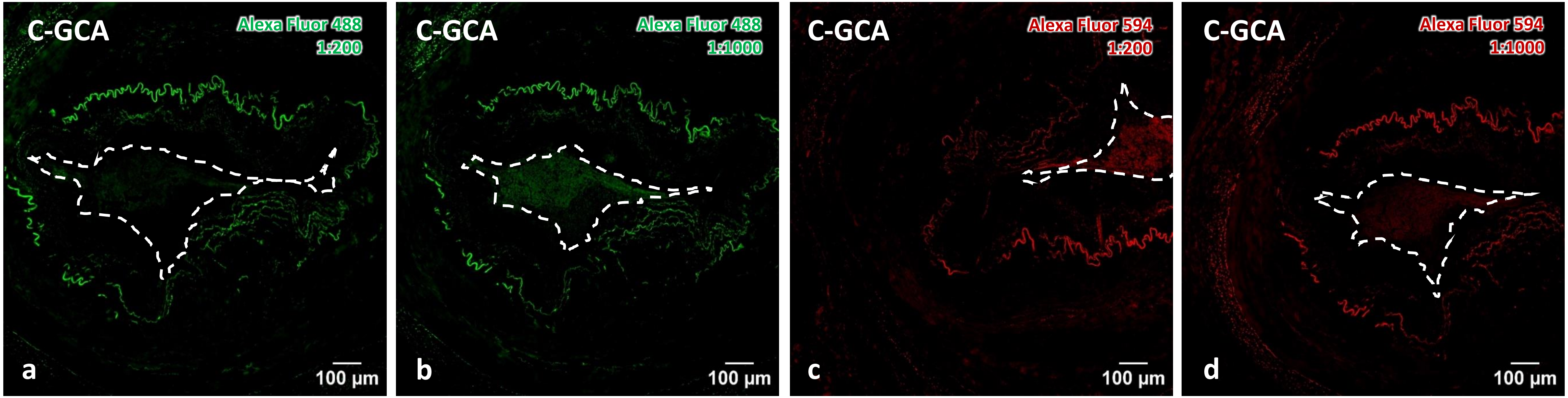

Figure S2: Staining results with secondary antibody at two different dilutions (1:200 and 1:1000), considered as „background staining“. **a, b**: Staining with goat anti-rabbit IgG, Alexa Fluor, Invitrogen, Cat. A-11034. **c, d**: Staining with goat anti-mouse IgG, Alexa Fluor, Invitrogen, Cat. A-11037.

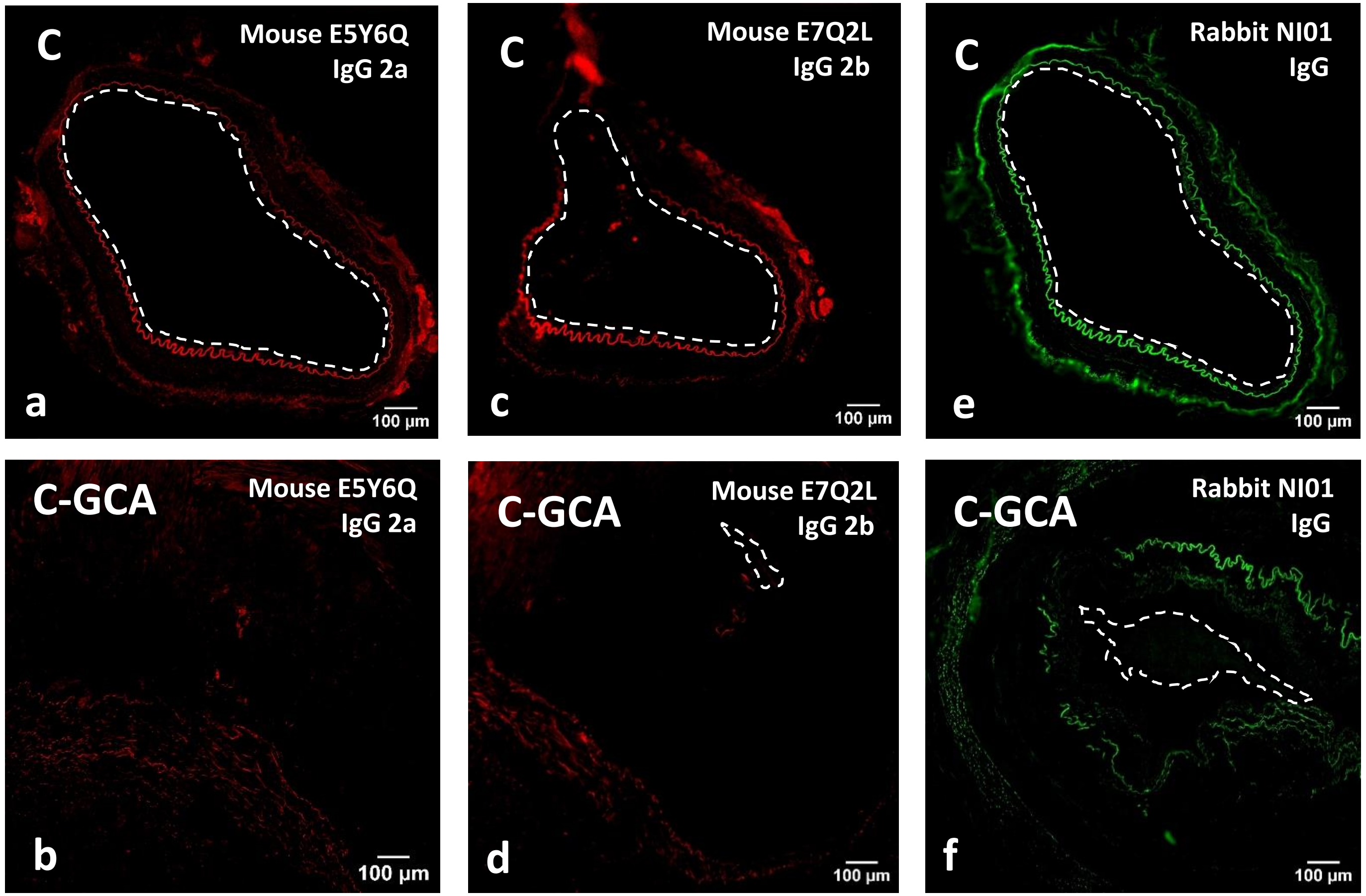

Figure S3: Staining of TAB samples from controls and C-GCA patients with isotype-antibodies and corresponding secondary antibodies. **a,b:** Staining with mouse Anti-IgG 2a antibody, Cell Signaling Tehcnology, Cat.61656. **c,d**: Staining with mouse Anti-IgG 2b antibody, Cell Signaling Technology, Cat.53484. **e,f**: Staining with rabbit Anti-IgG antibody, Sigma Aldrich, Cat.NI01.

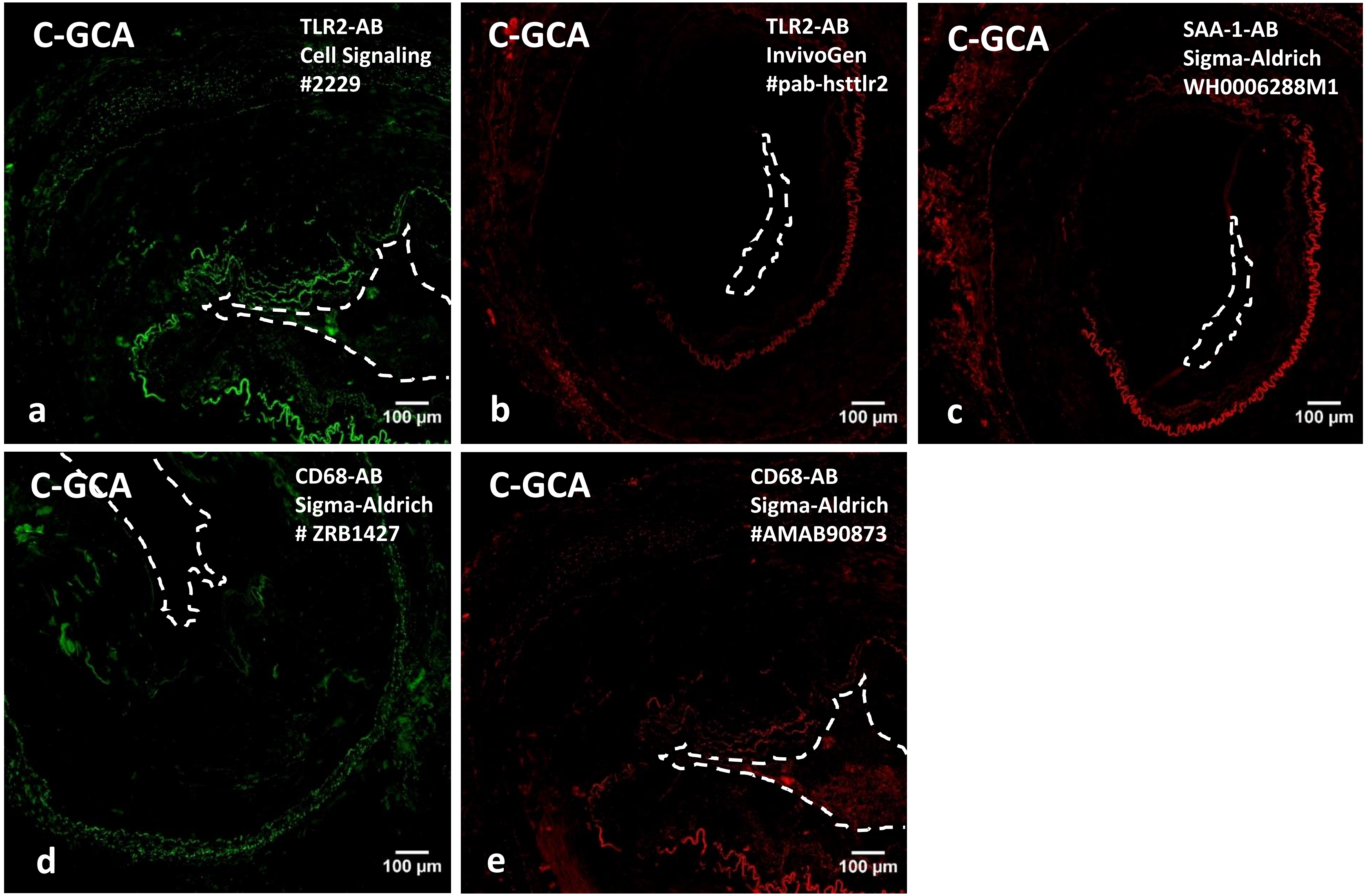

Figure S4: Staining results with various commercial antibodies in TAB samples from patients with C-GCA. Staining intensity is not different from figures Fig. S2, S3, and thus, it was considered negative. **a:** Staining with rabbit anti-TLR2 antibody, Cell Signaling Technology, Cat.2229. **b**: Staining with rat anti-TLR2 antibody, InvivoGen, Cat.pab-hsttlr2. **c**: Staining with mouse anti-SAA-1 antibody, Sigma Aldrich, Cat.WH0006288M1. **d**: Staining with rabbit anti-CD68 antibody, Sigma Aldrich, Cat.ZRB1427. **e**: Staining with mouse anti-CD68 antibody, Sigma Aldrich, Cat.AMAB90873.

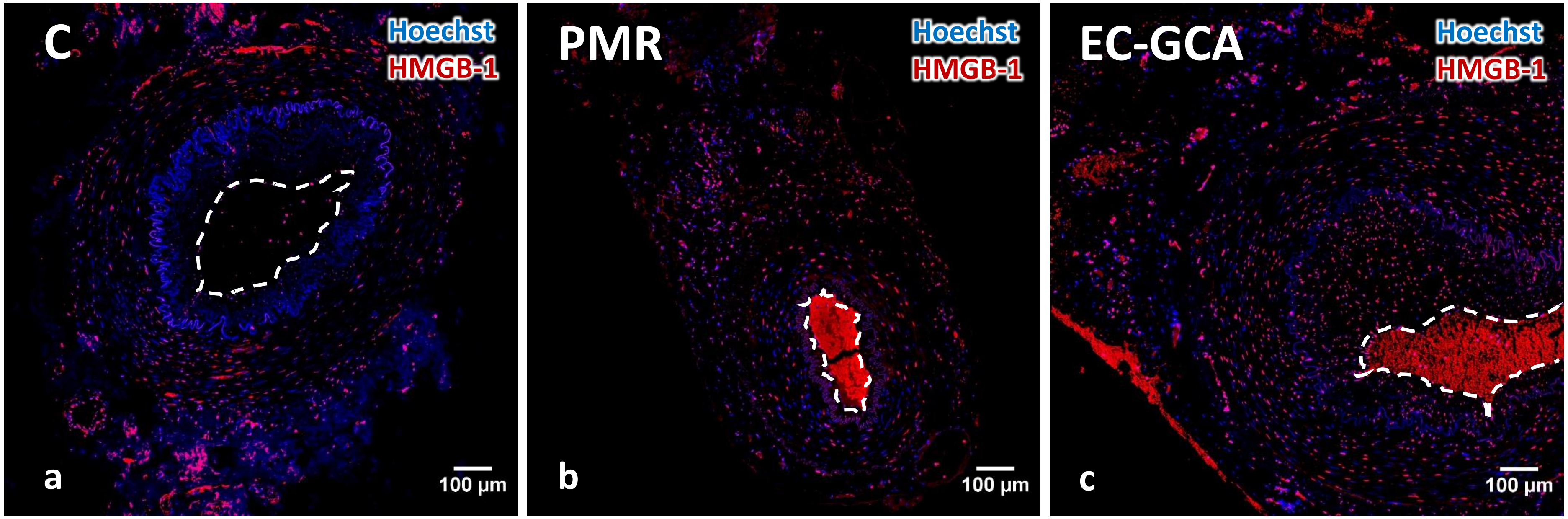

Figure S5: Double staining with anti-HMGB-1 (red color) and Hoechst (blue color) in TABs sections. **a**: Control. **b**: PMR. **c**: EC-GCA.

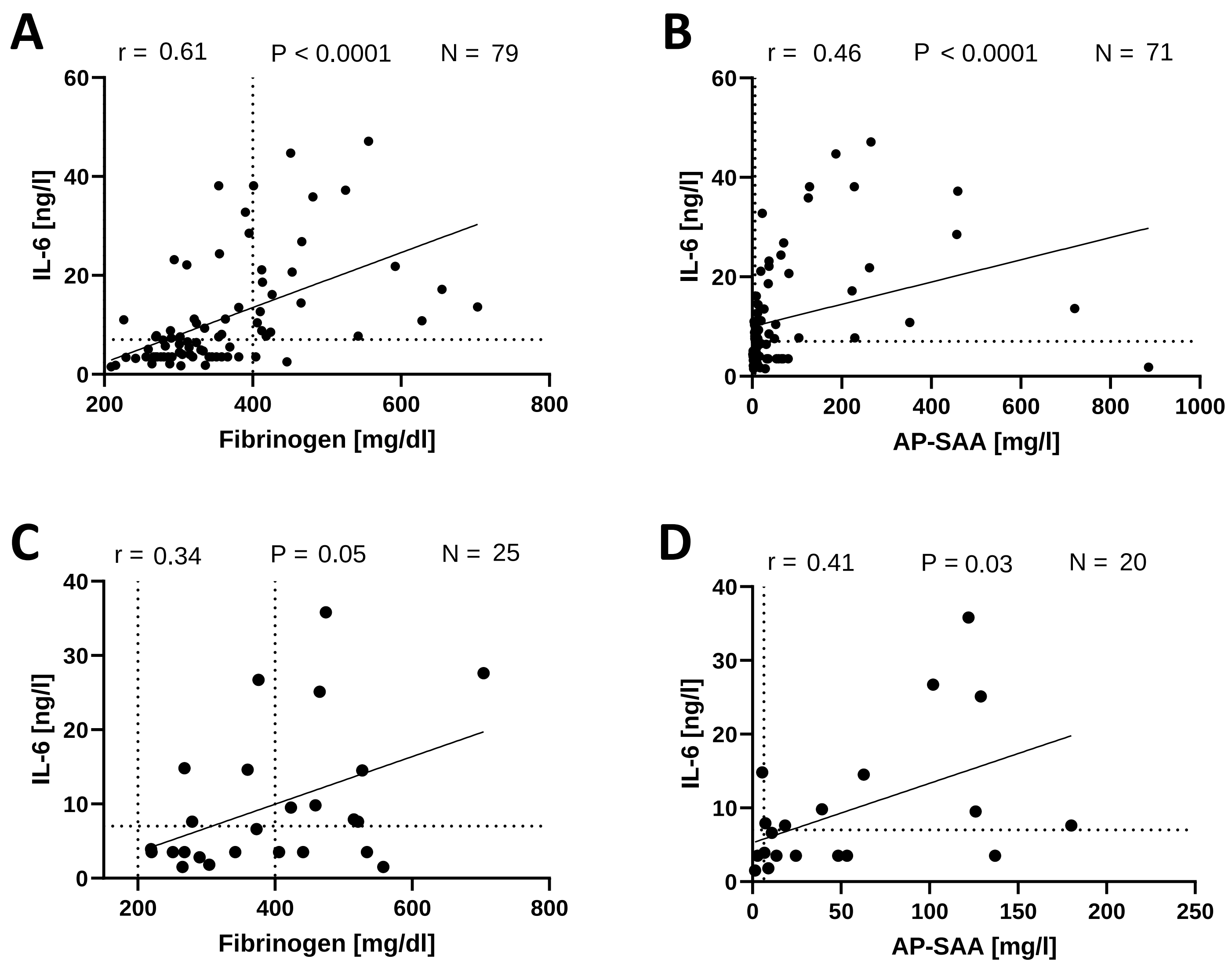

Figure S6: Association between the values of AP-SAA or Fibrinogen and IL-6 values of PMR (**A** and **B**) and GCA patients (**C** and **D**). r: Spearman´s rank correlation coefficient; P: p-values significant if p < 0.05; N: Number of patients; Dotted line: reference values.

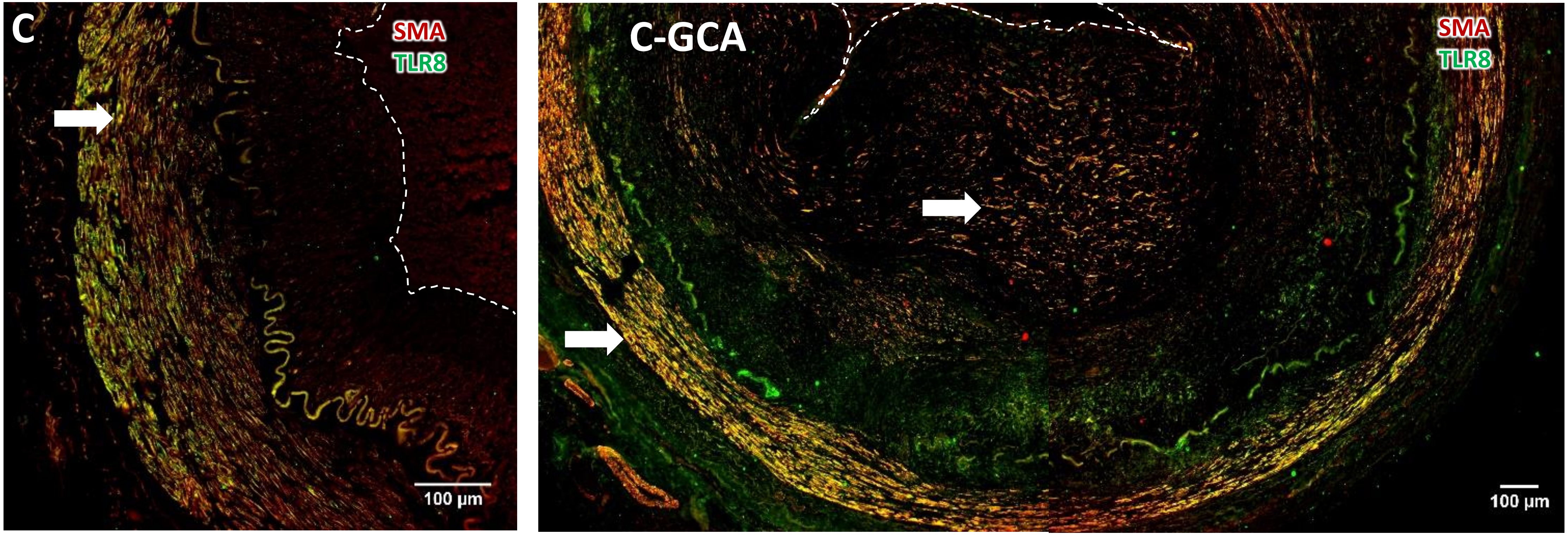

Figure S7: Expression of TLR8 and SMA in TABs of control and GCA samples.
TLR8 (green) and smooth muscle actin (SMA, red) staining.
White arrows: expression of TLR8 in smooth muscle cells in the tunica media (control) or tunica media and tunica intima (GCA).

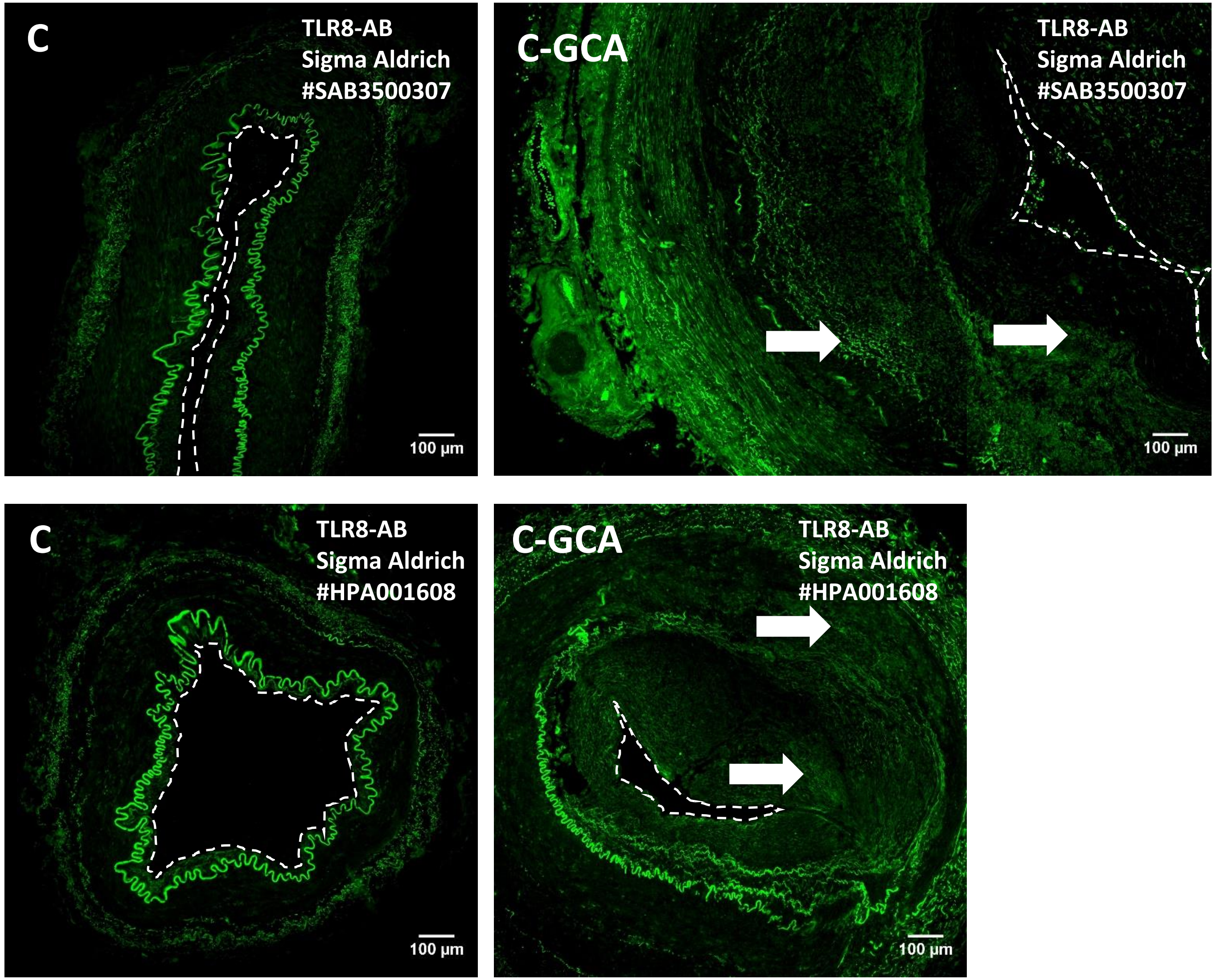

Figure S8: TLR8 expression detected with two different anti-TLR8 antibodies.
